# On the global CRISPR array behavior in class I systems

**DOI:** 10.1186/s13062-017-0193-2

**Published:** 2017-08-29

**Authors:** Alice Toms, Rodolphe Barrangou

**Affiliations:** 1 Bioinformatics Research Center, North Carolina State University, Raleigh, NC 27695 USA; 2 Center for Integrated Fungal Research, Department of Entomology and Plant Pathology, North Carolina State University, Raleigh, NC 27695 USA; 3 Department of Food, Bioprocessing and Nutrition Sciences, North Carolina State University, 400 Dan Allen Drive, Schaub Hall, Campus box 7624, Raleigh, NC 27695-7624 USA

## Abstract

**Background:**

Much effort is underway to build and upgrade databases and tools related to occurrence, diversity, and characterization of CRISPR-Cas systems. As microbial communities and their genome complements are unearthed, much emphasis has been placed on details of individual strains and model systems within the CRISPR-Cas classification, and that collection of information as a whole affords the opportunity to analyze CRISPR-Cas systems from a quantitative perspective to gain insight into distribution of CRISPR array sizes across the different classes, types and subtypes. CRISPR diversity, nomenclature, occurrence, and biological functions have generated a plethora of data that created a need to understand the size and distribution of these various systems to appreciate their features and complexity.

**Results:**

By utilizing a statistical framework and visual analytic techniques, we have been able to test several hypotheses about CRISPR loci in bacterial class I systems. Quantitatively, though CRISPR loci can expand to hundreds of spacers, the mean and median sizes are 40 and 25, respectively, reflecting rather modest acquisition and/or retention overall. Histograms uncovered that CRISPR array size displayed a parametric distribution, which was confirmed by a goodness-of fit test. Mapping the frequency of CRISPR loci on a standardized chromosome plot revealed that CRISPRs have a higher probability of occurring at clustered locations along the positive or negative strand. Lastly, when multiple arrays occur in a particular system, the size of a particular CRISPR array varies with its distance from the *cas* operon, reflecting acquisition and expansion biases.

**Conclusions:**

This study establishes that bacterial Class I CRISPR array size tends to follow a geometric distribution; these CRISPRs are not randomly distributed along the chromosome; and the CRISPR array closest to the *cas* genes is typically larger than loci in *trans*. Overall, we provide an analytical framework to understand the features and behavior of CRISPR-Cas systems through a quantitative lens.

**Reviewers:**

This article was reviewed by Eugene Koonin (NIH-NCBI) and Uri Gophna (Tel Aviv University).

**Electronic supplementary material:**

The online version of this article (doi:10.1186/s13062-017-0193-2) contains supplementary material, which is available to authorized users.

## Background

Clustered regularly interspaced short palindromic repeats (CRISPRs) and associated sequences (*cas*) function as the adaptive immune system in bacteria and archaea, to protect against phages and fend off plasmids [1]. Mechanistically, CRISPR-Cas systems provide DNA-encoded [1], RNA-mediated [2], and DNA-targeting [3, 4] of invasive DNA. In the past decade, CRISPR-Cas systems have been subjected to extensive studies to understand their features and characterize their functions [5]. As microbial communities and their genome complements are being determined across the globe, important biological and genomic features get characterized through in silico*,* in vitro, and in vivo analyses [6]. Though an increasing amount of bacterial genome data is available in Genbank, the subset of organisms that have been subjected to genome sequencing is unfortunately not equally representative across the phylogenetic tree, and displays a bias towards pathogenic species, justifiably. This well-documented bias can possibly influence survey-type studies, and may lead to conclusions that may or not be applicable throughout the tree of life. Much attention is currently dedicated to CRISPR-Cas systems given their important functional role in adaptive immunity, and their tremendous potential as genome editing molecular machines [7, 8]. Actually, CRISPR-based genome editing technologies have been rapidly democratized and yielded thousands of manuscripts in the last two years [9]. We are now at a point in time where we can analyze these systems qualitatively and quantitatively. Recently, much progress has been made regarding the classification and nomenclature of diverse CRISPR-Cas systems. In particular, it has been determined that these systems can be classified into two classes, based on the types and sequences of *cas* genes associated with CRISPR arrays [10]. Importantly, the genetic classification is also corresponding to their biochemical modes of action. Currently, Class I consists of Types I, III, and IV and Class II encompasses Types II, V, and VI [10–16]. CRISPR-Cas systems are diverse, genetically and mechanistically [17], and the relative occurrence of various types and subtypes is highly variable across phylogenetic groups in bacteria and archaea, notwithstanding their common origin [18, 19]. Nevertheless, there is only so much depth established to date across types and subtypes, and it has been repeatedly established that Class I systems are most abundant and widespread [10]. The availability of a larger dataset for Class I systems in general, and Type I systems in particular, enables us to assess a larger sample, as compared to Class 2 systems, which would be more prone to sample bias given the still limited availability of genomes that encode these loci. We thus elected to investigate the features of Class I systems, focusing on the widespread Type I and Type III CRISPR-Cas systems. Conveniently, this is also the most diverse set of systems and subsystems across CRISPR classes. Whereas Type II systems are arguably the most popular in the literature [9], their relative paucity compared to Type I systems limit the opportunities to quantitatively carry out statistical analyses of interest in this study. Furthermore, this is compounded by the fact that the large majority of studies of Class 2 systems are limited to the SpyCas9 (*Streptococcus pyogenes*) Type II-A subtype [7]. Likewise, with regards to phylogenetic sampling of the data, the bacterial kingdom is of special interest because proportionally it has less incomplete or ambiguous classification of its CRISPR-Cas systems and there is also a much larger number of strains and genomes to draw information from than the archaea kingdom, which has been subjected to a more limited extent of sequencing and characterization, though archaeal genomes tend to carry CRISPR-Cas systems more frequently [10]. For this study, much of the analyses performed are based on a combination of hand curation and computation of CRISPR array sizes and location/distribution to determine the association between documented CRISPR arrays and accompanying *cas* genes. Our primary objective was to carry out statistical analyses to investigate the distribution of Class I CRISPR arrays by size and genomic distribution, and carry out comparative analyses between types and subtypes.

## Results

### Distribution of CRISPR Array size

**Fig. 1:**
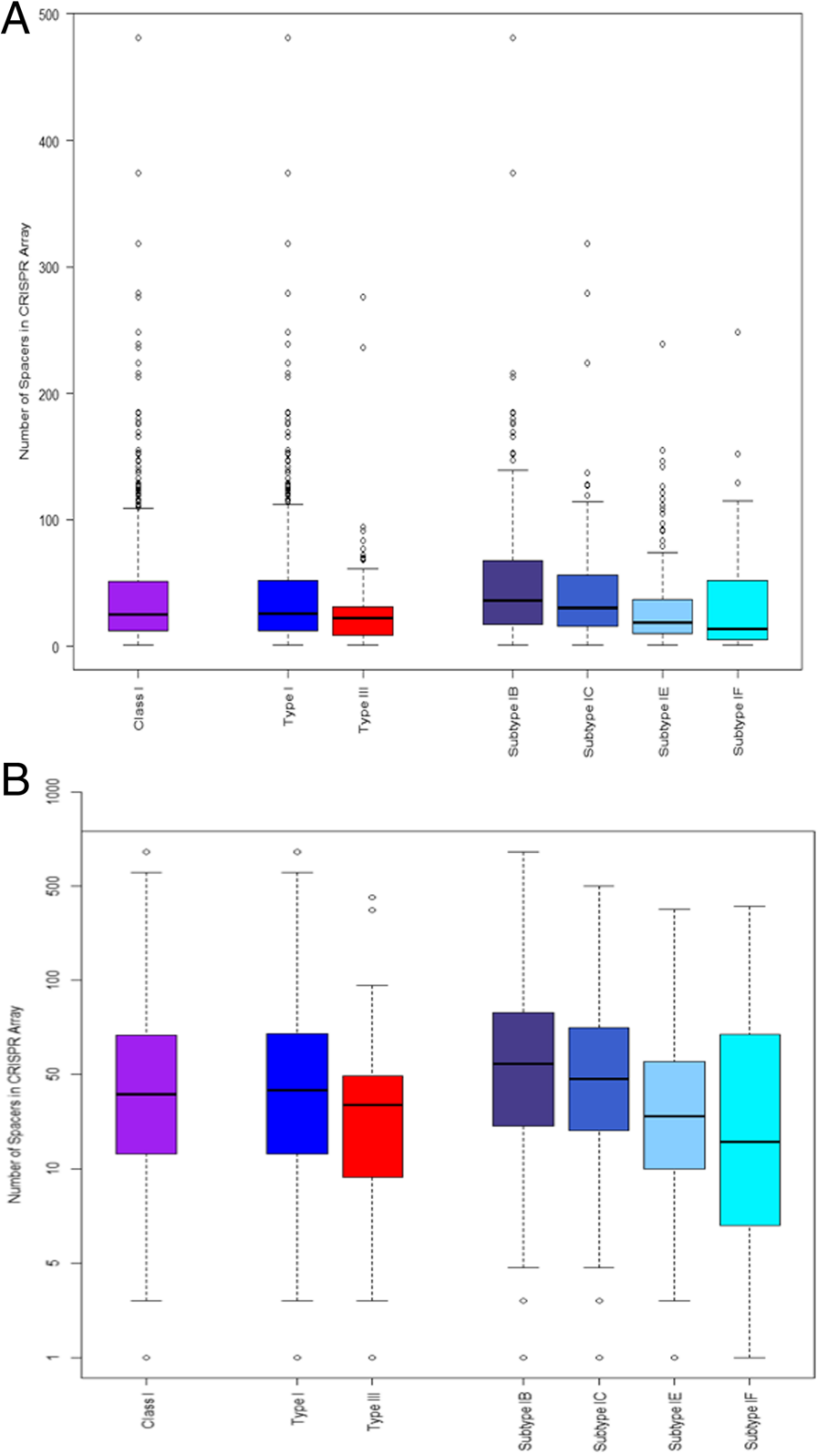
Array size across CRISPR-Cas types and subtypes. **a** Box plots of the number of spacers in a CRISPR array by class, type and subtype. **b** Boxplots of the number of spacers in a CRISPR array by class, type and subtype in a log 10 scale

Initially, descriptive statistics were performed to get a sense of the distribution of CRISPR array size of Class I CRISPR-Cas systems for bacterial chromosomes based on the numbers of spacers in an array. Figure 1 displays side-by-side boxplots while Table 1 outlines specific values for quartiles along with mean values when it comes to Class I, Type I, Type III, and subtypes I-B, I-C, I-E, and I-F. There is a general trend that mean values are between 30 and 54 spacers for this aggregated data, with an overall mean of 40 and median of 25. We also note that the median values range from 14 to 36 spacers, suggesting that the outliers, especially the maximum values, skewed our means to be larger, especially given outliers for which the number of CRISPR spacers reaches several hundreds. Our interest also turned to the statistically significant differences that may exist between Type I and Type III systems, as well as between the major four subtypes within Type I systems. We first tested the null hypothesis that there is not a difference between the means for Type I and Type III CRISPR array size. We immediately see from our descriptive statistics that Type I and Type III vary in sample size, so we performed a 2-sample t-test with unequal variances, as not to assume unequal variance. Our result showed a *p*-value of 0.098, and hence we fail to reject our null hypothesis and conclude that there indeed is no difference when it comes to the mean array sizes of Type I and Type III CRISPRs. Next, we decided to perform the Kruskal-Wallis test, to test our null hypothesis that the subtypes come from identical populations, without making any assumptions about the underlying populations. Our results indicated, with a *p*-value <0.001, that we reject our null hypothesis and thus conclude that there are statistically significant differences between the four subtypes, in that they do not come from identical populations.

**Table 1:** Summary statistics of CRISPR array size based on number of spacers in CRISPR-Cas Systems

	Mean	1st Quart	Median	3rd Quart		Systems
Class 1	40	12	25	51	481	811
Type I	41	12	26	52	481	708
B	54	17	36	68	481	188
C	43	16	30	56	318	164
E	30	11	19	37	239	226
F	33	5	14	52	248	91
Type III	33	9	22	31	276	103

**Fig. 2:**
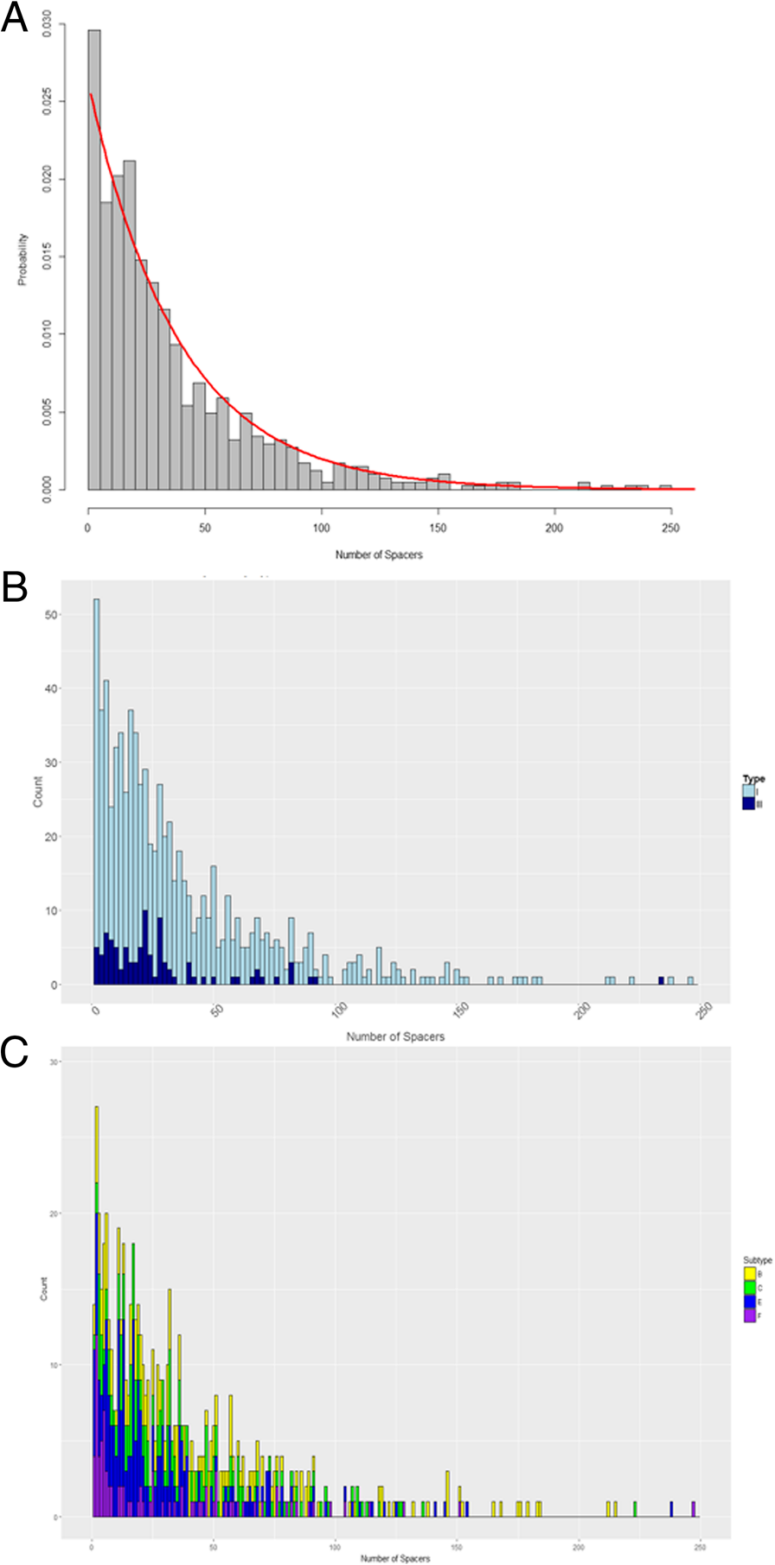
Distribution of CRISPR locus size. **a** Histogram of the distribution of Class I CRISPR locus size (number of spacers). **b** Histogram showing CRISPR array size by type. **c** Histogram showing CRISPR array distribution by select subtypes within Type I

**Fig. 3:**
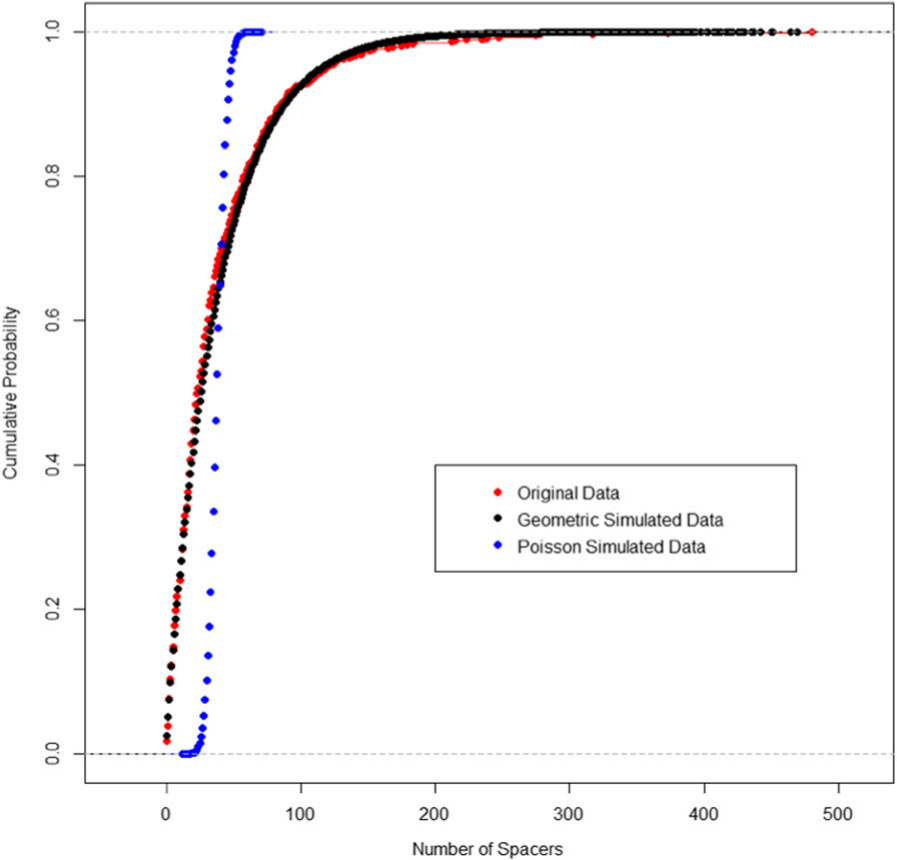
Fitting CRISPR array size distribution. Cumulative curve as a visual representation of the K-S test, showing that the geometric curve is a better fit than the competing alternative, the Poisson curve

A histogram was plotted for visual inspection of the observed values for Class I CRISPR array size displayed in Fig. 2. As mentioned before, the discrete values and right skewed graphic presented us with the possibility that array size followed either a geometric or Poisson distribution. After carefully considering the two candidate distributions for CRISPR array size of CRISPR-Cas systems in Class I bacterial chromosomes, it was found that the geometric distribution was the better fit compared to Poisson. The parameter estimates for geometric and Poisson respectively were 0.025 and 38.277. Both curves were plotted against the histogram and it was immediately apparent that the geometric curve was better suited. A histogram of the data with the fitted geometric curve was plotted as seen in Fig. 2a and subsequently broken up by type and subtype in Fig. 2b and c. Diagnostically, we conducted a K-S test and plotted a cumulative curve for the observed data and candidate distributions. We assessed two candidates, the Poisson and the Geometric distributions. The null hypothesis for the one-sample K-S test was that the sample data was drawn from a theoretical geometric distribution. The other null hypothesis was that the sample data was drawn from a theoretical Poisson distribution. The resulting *p*-value for the former null hypothesis was 0.048 and because the sample is so large with 811 observations, there is a lot of power to detect even some of the smallest differences. A probability plot was created that confirmed visually that the theoretical geometric distribution had fitted well to the sample data as seen in Fig. 3. Subsequently, this framework was used to also determine whether the remaining subsets of Class I also followed a geometric distribution. Overall, it was seen that generally they exhibited a similar behavior to Class I and they followed the geometric distribution as well. The estimated parameter values and *p*-values for fitting the geometric distribution to the sample data can be found in Table 2.

**Table 2:** Geometric Parameter Estimates and *P*-Value Results of Statistical Goodness of Fit Tests

	Maximum Likelihood Estimates: Geometric Parameter	Geometric Distr. For Array Size	CRISPR Locus: Uniform (+)	CRISPR Locus: Uniform (−)
Class I	0.025	0.048	0.000	0.000
Type I	0.025	0.238	0.000	0.000
B	0.019	0.821	0.002	0.000
C	0.023	0.143	0.115	0.014
E	0.034	0.153	0.000	0.000
F	0.030	0.001	0.495	0.754
Type III	0.031	0.029	0.906	0.000

Based on the statistical test performed and the investigation of a visual representation of those tests, it was concluded that CRISPR array sizes tend to follow a geometric distribution.

### Location of CRISPRs along chromosome

**Fig. 4:**
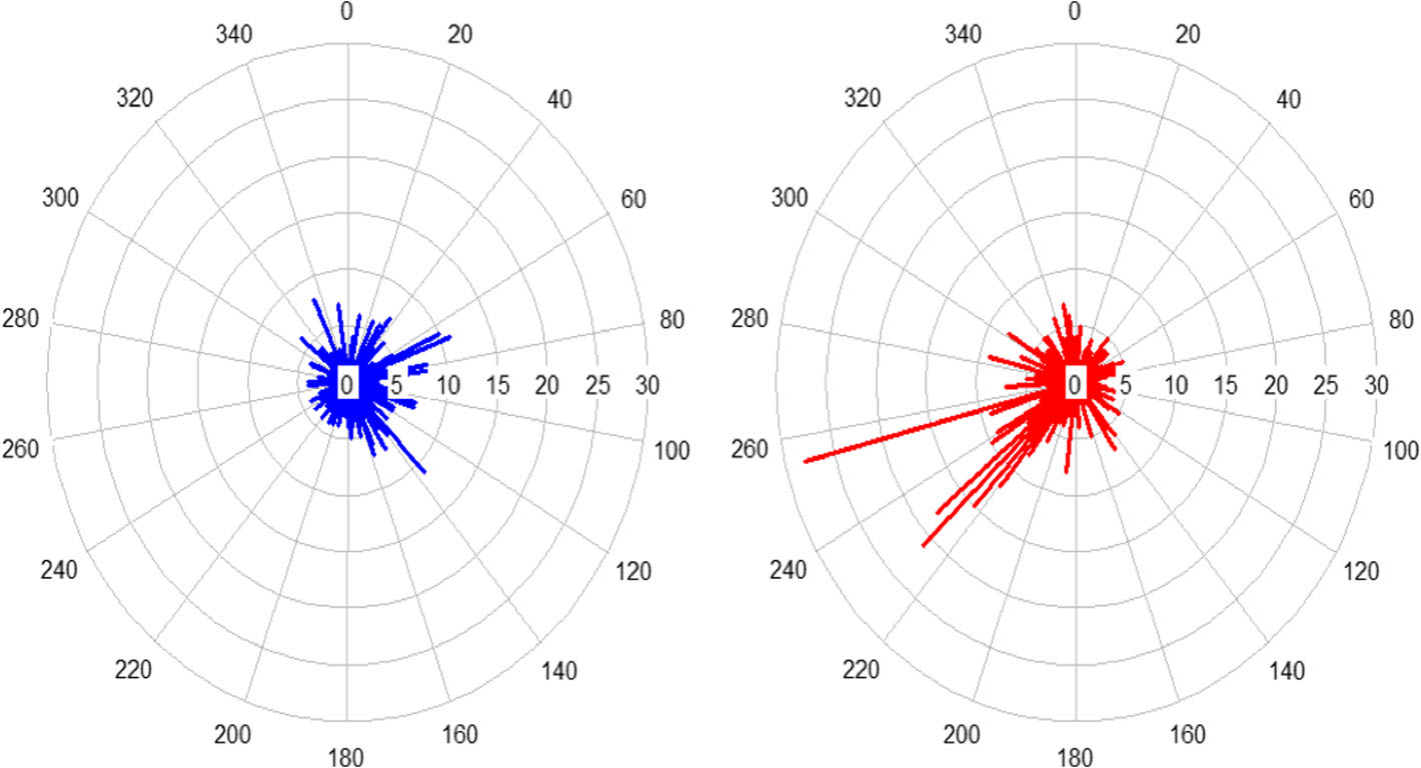
Genomic distribution of CRIPSR loci on chromosomes. Polar plot of the distribution of Class I CRISPR-Cas systems on the positive (left) and negative (right) strands of chromosomes

Initial examination of the polar plots showed that CRISPR loci systems had a tendency to be not-randomly distributed on bacterial chromosomes. Indeed, there appeared to be biases towards specific chromosomal locations. Generally, it was immediately apparent that certain areas of the chromosome tend to have larger concentrations of where CRISPRs occur versus other areas when standardizing the relative location of these systems. When looking at Class I overall, there is a large number of occurrences of CRISPRs beginning at the region between 200 and 240 degrees on the negative strand in the polar plot on the right of Fig. 4 and to a lesser degree, more occurrences between 60 and 120 degrees on the positive strand of the polar plots as seen on the left in Fig. 4. As the plots are further subdivided into the types and subtypes for the positive and negative strands, overall certain areas continue to be more pronounced. A formal test was conducted to determine whether the distribution of CRISPR arrays follows a uniform distribution, in which CRISPRs are just as likely to occur in relatively the same frequency anywhere along the chromosome. The null hypothesis would be that the CRISPRs followed a uniform distribution and a low *p*-value rejects this hypothesis. These results are displayed in Table 2. K-S tests performed generally exhibited low *p*-values particularly when over 200 observations were present for the test. This is a reminder that in such analyses, it is important to encompass enough data points as to reach a power threshold enabling such statistical analyses, which we were solely able to achieve for Class I systems. Some of the smaller subsets such as subtype I-F and Type III had larger *p*-values, but with too few observations to conduct this test. Overall, it was concluded that based on the results of the statistical tests and polar plots that CRISPRs do not generally begin randomly along the chromosome but instead have certain concentrated peaks (see Additional files 1 and 2). Mechanistically, we speculate that this may be correlated to genome-wide processes that could play critical roles in the expansion and maintenance of CRISPR arrays, as well as biochemical processes involved in the new spacer acquisition step during CRISPR immunization, such as chi sites, chromosomal replication, and DNA repair processes [20]. For instance, it is possible that selective pressure may incentivize the minimization of delays in replication of the leading strand, and select for the absence of collisions and stereo-physical hindrance between the replication machinery and the Cas proteins at the boundaries of CRISPR arrays. This may be most applicable to the leader end of CRISPR arrays, where integration occurs and transcription of pre-crRNAs and processing of crRNAs is initiated, and the highest. Furthermore, there are likely two structural challenges inherent to CRISPR loci and CRISPR repeats, since the former contains heavily repeated stretches of DNA (by nature), and the latter is partially palindromic. These repeats and secondary structure therefore pose structural challenges that may be further compounded by multiple interactions with various Cas proteins involved in array maintenance, replication, and expansion. Thus, it is possible that biases in location and orientation of CRISPR loci may reflect selection for the minimization of structural and processing disruptions.

### Analysis of the correlation between CRISPR array size and the number of loci in a chromosome (or proportion of largest CRISPRs associated with CRISPR-Cas systems and number of CRISPRs in a genome)

**Fig. 5:**
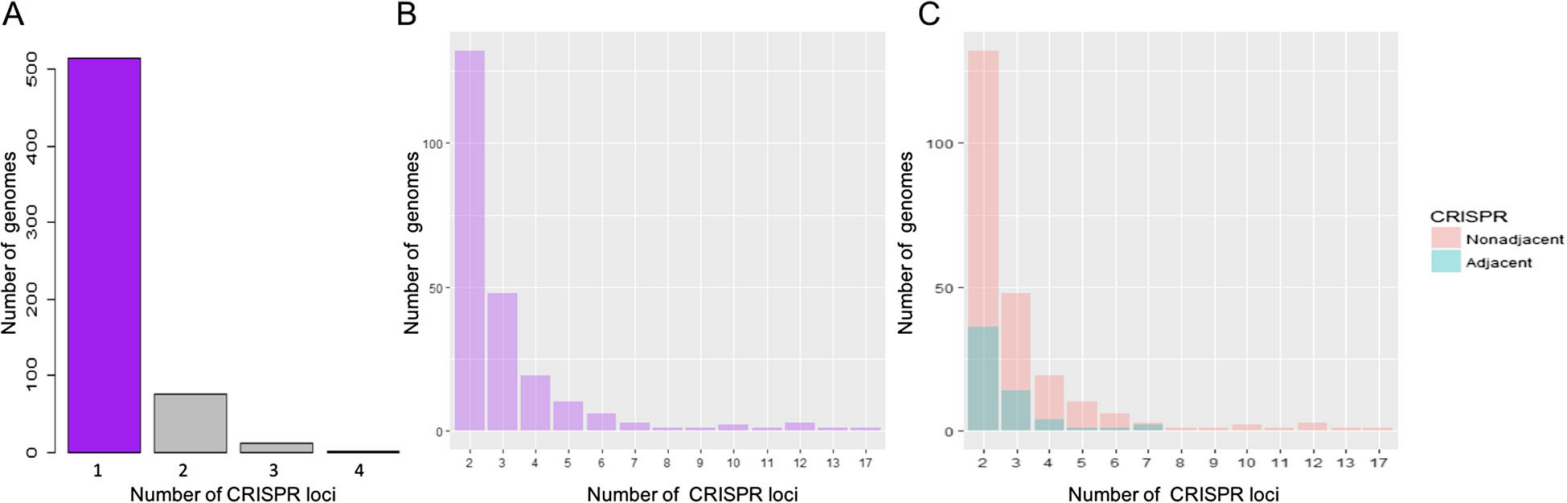
CRISPR locus occurrence and position relative to *cas* operons. **a** Histogram representing the frequency of genomes carrying the specified number of CRISPR loci. Histograms representing the relationship of the proportion of CRISPR arrays that are adjacent to the *cas* operon. **b** Number of CRISPR arrays encoded on a given chromosome. **c** Location of the CRISPR arrays relative to the *cas* operon

In our data set, it was found that 82% of bacterial Class I CRISPR-Cas systems had only one set of *cas* genes as show in Fig. 5a. We next investigated patterns that may occur when multiple arrays occur in one chromosome. After isolating all systems that had only one set of *cas* genes, the rank correlation statistic was calculated for Class I and the two major types for the relationship between the number of spacers in the CRISPR array that is closest to the *cas* genes and the number of arrays in the genome. Spearman’s rho can result in values along the spectrum between −1 and 1 where higher values indicates a stronger association between the two variables. Class I had a value of −0.85, Type I had a value of −0.79, and Type III had a value of −0.75. These values showed a strong association in the manner that as you increased the number of CRISPRs present in a genome that had only one CRISPR-Cas system, the CRISPR closest to the set of *cas* genes was less likely to be the largest CRISPR in the genome. A visual representation for Class I can be seen in Fig. 5b.

Therefore, it was concluded that CRISPRs tend to be larger when they are in closer proximity to a set of *cas* genes but as you increase the number of CRISPRs on a chromosome, then the probability of the largest CRISPRs being associated with those *cas* genes tends to decrease for chromosomes with a single set of *cas* genes. Mechanistically, we hypothesize that this enables the host to control the expansion of one given locus, which would be advantageous transcriptionally, by having the ability to both initiate transcription from multiple locations, and also limit the size of primary CRISPR transcripts.

## Discussion

CRISPR-Cas data genesis has reached a point where statistical analyses can be performed to examine these intriguing loci and investigate quantitative behaviors of CRISPR arrays with regards to size and distribution, for Class I systems in bacterial genomes. Recent efforts in CRISPR-Cas systems classification, in combination with the availability of CRISPR and *cas* databases are affording new opportunities to gain insight into these systems. In particular, we can now examine the number of spacers in CRISPR arrays in Class I systems, and also between the major types (Type I and Type III) and even between well-represented subtypes (I-B, I-C, I-E, and I-F). Previous research focused on knowledge of individual strains but now the collective gives rise to the ability to examine CRISPR-Cas systems from a quantitative perspective.

It is important to note that these results were derived from a sample dataset. CRISPR-Cas systems are not ubiquitous in nature, and occur in approximately 46% of sequenced microbial genomes, and 90% of available archaeal genomes. Importantly, there are notable biases in the genomic information available to date, as it is not an even sampling of natural microbial diversity, and it also heavily represents pathogenic bacteria. In some instances, it also over-represented and redundantly encompasses multiple isolates and strains of select pathogenic species. Thus, these numbers may not reflect the actual natural distribution patterns of CRISPR-Cas systems, which explains the discrepancy observed between the aforementioned frequencies of occurrence in sequences genomes vs. recently determined metagenomes [6]. Furthermore, when bacteria carry CRISPR-Cas, they typically only include one system and mostly one array. Future studies should investigate whether our findings also apply to Class 2 CRISPR-Cas systems as more data becomes available. This will also afford the opportunity to compare and contrast these two classes. Though Class 2 systems may be currently under-represented, and may actually occur at low frequencies, metagenomics surveys underway, and sampling expeditions aimed at finding new microbial diversity may encompass new Class 2 systems, especially given the desire to find new single effector nucleases. Furthermore, it will be intriguing to see what happens in organisms in which multiple CRISPR-Cas systems from both classes and multiple Types occur (i.e. cases such as *Streptococcus thermophilus*). Of course, as novel genomes become available, and novel CRISPR-Cas systems get identified and characterized, new features may come into play (such as the universality, or not, of *cas1* genes, and other markers and signature proteins). Furthermore, more questions may arise as the structure and function of CRISPR-Cas systems continue to be determined.

Also, once more data is available for archaea, it will be intriguing to investigate these peculiar systems, which tends to both be larger, and encompass multiple split loci within one chromosome.

It is also important to note that there is misconception or even misperception regarding the ease with which CRISPR-Cas systems are identified, categorized, and annotated. Indeed, there is a need to develop novel bioinformatics pipelines to streamline these processes, and optimize each of these steps dynamically as the classification evolves. Regardless, there is always a need for manual curation of the data currently available, to ensure these peculiar and idiosyncratic systems are properly annotated and categorized, which is increasingly challenging given their diversity and the few features they share in common. As always, data integrity is just an integral component as the quantitative and statistical analyses performed on the data itself. Overall, our findings lay a foundation for quantitative analyses of CRISPR-Cas systems, and open new avenues of investigating of these fantastic and valuable molecular machines.

## Conclusions

A statistical framework and visual analytics were utilized to examine CRISPR array sizes based on the number of spacers in a locus. Results showed that generally, array size follows a geometric distribution pattern after performing a goodness of fit test using the K-S test. Furthermore, we found that the starting position of CRISPRS does not occur uniformly along a chromosome. Lastly, we observed that when it comes to class and type, the CRISPR locus flanking the *cas* genes on a chromosome tended to be largest until you start increasing the number of CRISPRs in the genome based on the values calculated from Spearman’s rho. Essentially, we found a strong association between CRISPR size and distance from a set of *cas* genes in genomes where only one set of *cas* genes was present.

## Methods

To manage, filter, and merge data sets necessary for analysis, R software [21] was the primary resource. Additionally, it provided tools within its packages to perform statistical analyses and support a visual framework of the results as well.

### Data integration

After importing the *cas* data set from the 2015 CRIPSR-Cas classification revision study [10] using the readxl package [22], it was systematically explored with the help of the plyr package [23] along with the base package functions to identify and subset the *cas1* genes. Based on the structure of this data set, alongside each *cas1* gene, a specific system type and subtype was listed to identify the classification of the consecutive rows of *cas* genes for a given system. It is important to note that some genomes are listed more than once which means some have more than one system. Indeed, multiple CRISPR-Cas systems occur in genomes, across classes, types and subtypes. Consequently, our final data set contains strictly Class I systems and even more specifically, Type I and Type III systems. We excluded the uncharacterized and rarely occurring Type IV systems, and did not investigate arrays that were not associated with *cas1* markers.

The other data set imported was made up of CRISPR arrays identified and currently contained in an online database created by Grissa, Vergnaud and Pourcel [24] and is publicly available at http://crispr.i2bc.paris-saclay.fr/crispr/. We specifically focused on data from 811 complete bacterial genomes available in the July 2016 release, that carried a CRISPR locus, excluding those identified as “questionable structures”, with exclusion of loci identified on plasmids (as to solely focus on chromosomal features). This data was merged with the *cas* data [10] based on Accession ID to identify which CRISPRs are associated with which *cas* genes as well as take into account inactive CRISPR-Cas systems in the analysis. The last step was to segregate the CRISPR-Cas systems based on whether they were on the positive or negative strand of a chromosome as part of another analysis based on the location of CRISPR arrays. For CRISPR-Cas analyses, base pair distances between the *cas* genes and multiple CRISPR arrays were calculated and the CRISPR with the least distance from the as genes was chosen. We operated under the assumption that each set of *cas* genes was associated with one CRISPR. The final data set contained CRISPR-Cas genomes of Type I and Type III systems of Class I systems and were located entirely on bacterial chromosomes.

Throughout the process, GenBank hosted by NCBI was referenced repeatedly for verification of and details specific to mentioned organisms [25] and this information is publicly available at http://www.ncbi.nlm.nih.gov/genbank/. Curating the data for accuracy was a part of the process before any and all analyses were performed.

### Statistical Framework & Visual Analytics

A set of statistical and visual tools were implemented to answer each of the research questions with an emphasis on various packages in *R.* Major analyses were done on Class I genomes as well as Type I, Type III, and the following major subtypes of Type I: B, C, E, and F. We explicitly selected a discrete distribution to understand the behavior of the size of CRISPR arrays because they have a tendency to add spacers one at a time, and remove them often times in small blocks of 2–5 units, though this has only been shown in a select few systems. The repeat-spacer unit within CRISPR arrays is a core feature of these loci, and constitute a countable positive integer unit which is mathematically discrete.

### CRISPR Array size distribution of CRISPR-Cas systems

To investigate the distribution of CRISPR array size of active CRISPRs that belong to CRISPR-Cas systems, candidate distributions were fitted to the observed data of a histogram [26]. Poisson and geometric distributions were initially considered because both were discrete and right-skewed [27], which was consistent with the plotted histogram of CRISPR array size from the data set. The main difference between the geometric and Poisson distributions is that the geometric distribution is known to count the number of trials needed to get one success while Poisson counts the number of occurrences of rare events in a fixed interval of time where the number of trials is unknown [27].

Maximum likelihood estimation method [28] was used to determine the parameter estimates for the single-parameter candidate distributions and it is displayed in the following as the likelihood of the parameter given as a function of the joint density for parameter θ for n observations of x [27]:

1 $$ \mathrm{lik}\left(\theta \right)=f\left({x}_1,{x}_2,\dots, {x}_n|\theta \right) $$

Using this information, the likelihood can also be rewritten in more detail as the following for *i*th observation in *n* sample size [27]:

2 $$ {l}^{\prime}\left(\theta \right)=\frac{\partial l}{\partial \theta}\left(\sum_i^n\mathit{\ln}\left[f\left({X}_i|\theta \right)\right]\right) $$

It is not sufficient to only fit a distribution because it may not turn out to be a proper fit to explain the quantitative behavior of array size. Nonparametric goodness-of-fit was the next step for comparing the data against the proposed distribution [29]. Generally, the Kolmogorov-Smirnov (K-S) test statistic has the following form where *F*(*x*) is the cumulative function for the data set and theoretical distribution to test the null hypothesis that the data does follow the specified theoretical distribution:

3 $$ D=\sup \left|{F}_0(x)-{F}_{data}(x)\right| $$

With the use of a modified discrete K-S test [29], the test can be properly applied to compare the candidate distributions with the observed array size. Furthermore, these results can also be displayed by means of a plot of the relative cumulative curves overlaid in one graphic for visual inspection of fit.

A powerful graphical tool for assessing the fit of data to a theoretical distribution is a probability plot [27]. Depending on sample size, it can be difficult to give a fair significance level at which to consider a *p*-value to be statistically significant and hence to combat the problem, this qualitative approach is essential. Probability plots depend on values called order statistics shown here with *j*th observation and *n* total observations [27]:

4 $$ E\left({X}_{(j)}\right)=\frac{j}{n+1} $$

It was hypothesized that the data may follow a geometric distribution by performing a Kolmorgorov-Smirnov test once the parameters are estimated for the proposed distributions.

Subsequently, additional histograms were created using reshape2 and ggplot2 R packages that showed the differentiation between Type I and II as well as the different subtypes within Type I when it came to visually examining their behavior for how closely they followed the geometric distribution [30, 31].

### Distribution of CRISPRs along chromosome of CRISPR-Cas systems

In determining if CRISPRs of CRISPR-Cas systems are non-randomly distributed along the chromosome, it was necessary to plot the positive and negative strands in addition to conducting a statistical analysis. Because CRISPRs can theoretically begin anywhere on a chromosome and different genomes can have different chromosome sizes due to different numbers of total base pairs, standardization became a way to compare genomes within and between system types and subtypes:

5 $$ \mathrm{Location}={\left(\frac{Locus Start}{Chromosome Size}\right)}^{\ast }360 $$

Polar plots were the initial diagnostic tool for plotting CRISPRs along a chromosome [32]. Essentially, the circular version of a histogram not only plotted where a CRISPR is found but also often it occurred at that location because the standardization rounded to the nearest integer.

Essentially, we wanted to see if the CRISPRs are just as likely to occur anywhere along a chromosome. A K-S test was performed and a probability plot was created to assess whether the observed CRISPR data was uniformly distributed along a chromosome.

It was hypothesized that the starting location of CRISPR arrays of CRISPR-Cas systems followed a uniform distribution.

### Relationship of the size of an CRISPR, its distance from *cas* genes, and the number of CRISPRs in a chromosome

Because both an active and inactive CRISPR has a repeat spacer array, we can examine the relationship between the size of a CRISPR regardless of whether it is associated with a CRISPR-Cas system and its distance from a set of *cas* genes.

A histogram can plot the number of CRISPR-Cas systems against the number of genomes so that we can subset the data set where the largest number of genomes occur for a specific number of CRISPR-Cas systems in a genome. A secondary histogram can display an aggregation of the former histogram to show the number of CRISPRs in a genome against the number of total genomes and the number of genomes where the CRISPR associated with the CRISPR-Cas system is the largest. Furthermore, we can find the correlation between the proportion of genomes where the largest CRISPR is the CRISPR associated with the CRISPR-Cas system and the number of CRISPRs in a genome. Using a special case of the Pearson correlation, we can determine the Spearman rank coefficient or Spearman rho coefficient to measure how strong of a correlation exists between the ranked versions of those two variables. This non-parametric method is also resistant to outliers which can be helpful in our case when a small number of genomes have a fairly large number of CRISPRs compared to the large majority of other genomes. The calculation of rho is determined by *N* ranks where *D*_*i*_ is the difference between the ranks of our two variables of interest for each *i*th case [33]:

6 $$ {r}_s=1-\frac{6{\sum}_i{D}_i^2}{N\left({N}^2-1\right)} $$

Spearman’s rho can be easily calculated with the use of software, in this case R provides the cor.test function as part of their stats package [21].

It was hypothesized that CRISPR arrays of CRISPR-Cas systems tend to be larger than CRISPRs further away from the *cas* genes on the same chromosome when there is a single set of *cas* genes on the chromosome.

## Reviewer comments

### Reviewer’s report 1: Eugene Koonin (NIH, NCBI)

Toms and Barrangou report bulk statistics on CRISPR arrays for class I CRISPR-Cas systems. Although the paper as such does not report genuine biological insights, these are useful data, and as the authors point out at the end of the Abstract, it is a step towards creating an analytical framework for quantitative analysis of CRISPR arrays. Thus, overall, this is useful work. However, I do have certain concerns regarding the methods, conclusions and presentation that are detailed below.

Authors’ response: *We thank the reviewer for their assessment of our work, and value ascribed to creating an analytical framework for quantitative analysis of CRISPR arrays. Indeed, there is a gap in quantitative analyses of CRISPR-Cas systems across a range of systems, since most studies have focused on select genus-species combinations, or particular CRISPR-Cas subtypes. We note the raised issues about methods, conclusions and presentations and have made edits, corrections, and additions as outlined below*.

#### Recommendations

My principal concern is quite technical. Clearly, any statistics over large collections of genomes can be significantly biased, to the point of being meaningless, by the effects of non-independence in the data. Because all genomes are connected by a tree, whereas genome sequencing so far had been strongly non-random, with numerous strains sequenced for only a few species and many species for only a few genera, this is a serious concern in microbial genomics.

Authors’ response: *We agree with the reviewer that indeed, there is a well-documented bias in how genome sequencing in microbes has been selectively focused on a subset of (mostly pathogenic) species, and has yielded bias sampling across the phylogenetic tree. We now acknowledge, disclose and mention this in our introduction section (see new text inserted lines 47–48). “Though an increasing amount of bacterial genome data is available in Genbank, the subset of organisms that have been subjected to genome sequencing is unfortunately not equally representative across the phylogenetic tree, and displays a bias towards pathogenic species, justifiably. This well-documented bias can possibly influence survey-type studies, and may lead to conclusions that may or not be applicable throughout the tree of life”*.

Granted, CRISPR-cas loci are highly variable, even between closely related isolates, so the problem in this case could be less severe than in others. Still, I think it is important for this kind of analysis to be clear about the way non-independence between genomes was taken into account or why it is unimportant if it was not. The significance and even validity of all results could be affected by this source of bias.

Authors’ response: *Again, we agree with the reviewer. Though statistical analyses typically hinge on independence between observations, or in this case, available complete genome sequences, it is important to note that we now acknowledge the absence of strict independence in our dataset. Importantly, we did not account for this non-random bias in our analyses, but this is why we limited our study to Type I systems, and focused specifically on select subtypes that encompass a larger and more diverse sample size. We discuss this in an added section inserted line 61 in the revised text. Notably, the relatively shallow dataset available for class 2 would preclude us from overcoming this bias. The following text was added: “The availability of a larger dataset for Class I systems in general, and Type I systems in particular, enables us to assess a larger sample, as compared to Class 2 systems, which would be more prone to sample bias given the still limited availability of genomes that encode these loci. We thus elected to investigate the features of Class I systems, focusing on the widespread Type I and Type III CRISPR-Cas systems. Conveniently, this is also the most diverse set of systems and subsystems across CRISPR classes.”*

Along the same lines but more with respect to presentation: I think the data, i.e. the genomes that were analyzed (or not analyzed) have to be described explicitly.

Authors’ response: *We now provide details in the “data integration” sub-section within the methods section (new text inserted line 256: “We specifically focused on data from 811 complete bacterial genomes available in the July 2016 release, that carried a CRISPR locus, excluding those identified as “questionable structures”, with exclusion of loci identified on plasmids (as to solely focus on chromosomal features.)”), and state that we used all complete bacterial genomes (N = 811) that were available at the CRISPRdb website (for which the url is inserted into the text) and specify the date (July1st 2016) at which the dataset was used. We now explain how we focused our efforts on analyzing the genomes in which a CRISPR locus had been putatively identified, excluding those categorized as “questionable structures”, with exclusion of CRISPR loci identified on plasmids (we solely included chromosomal features). Furthermore, we cross-referenced these with the curated cas gene dataset featured in the Makarova* et al.*, 2015* [10] *milestone reference, which importantly ascribed class, type and subtype, enabling us to confidently investigate a validated subset. If the reviewer and editor think this would be valuable to the readership, we could certainly provide a document with the genome information and details to post as supplementary material*.

Turning now to the results of this work, I can accept that geometric distribution is a better fit for the distribution of array lengths than Poisson distribution. It is not entirely clear for me why a discrete distribution was chosen in the first place (‘visual’ impression of the data is hardly a strong argument). Then, I have to admit that I am not sure I can make any conclusions from the observation that the geometric distribution wins over Poisson. The authors do not offer any insightful ideas on this, and I am afraid that would be difficult to do. Generally, I believe that such a straightforward comparison is not a particularly informative way to analyze distributions of CRISPR array lengths (or any other genomic features). It would be much more satisfying to examine different mathematical models of array growth (different variants of birth-and-death models, I think) and then fit different functions. In that manner, we might have some biological insights. The way the results are presented in the paper, we do not.

Authors’ response: *Indeed, the data and results clearly support the conclusion that a geometric distribution is a better fit than Poisson. In regards to the data, we now state that “we explicitly selected a discrete distribution to understand the behavior of the size of CRISPR array because they have a tendency to add spacers one at a time, and remove them often times in small blocks of 2-5 units, though this has only been shown in a select few systems. The repeat-spacer unit within CRISPR arrays is a core feature of these loci, and constitute a countable positive integer unit which is mathematically discrete.” We inserted text to that effect in the “statistical frameworks and visual analytics section”, lines 275–279*.

The apparent non-randomness of the CRISPR loci distribution on the chromosomes is a potentially interesting observation. Again, however, the authors offer no biological interpretation. Any speculation at all?

Authors’ response: *We wholeheartedly agree with the reviewer that this is perhaps the most novel, interesting and unexpected finding in our study. Interestingly, we had to repeat this analysis a few times to ensure the veracity of this observation. This was also brought up by the other reviewer and we now include a dedicated paragraph in the discussion section to speculate as to why this pattern may occur, and prompt the readership to investigate this phenomenon. Recent studies about CRISPR spacer integration processes and interplay between genomic features and CRISPR loci (replication forks, chi sites and more) are now discussed. (see lines 157–167).*

There are pertinent questions that (I think) could have been easily addressed in this work, but currently are not. Why analyze Class 1 only? I think quantitative comparison with Class 2 would be interesting. If there is a good reason not to do it, better to explain.

Authors’ response: *We agree with the reviewer that an all-encompassing Class 1 and Class 2 analysis would have been desirable in terms of thoroughness and completeness, especially given the current level of interest in Class 2 systems, with the intriguing opportunity to compare and contrast findings between these two main classes, but there were two challenges that precluded us from doing so: firstly, the shallow and even more biased sampling of Class 2 types, which precluded us from reaching high enough numbers to have the statistical power needed; secondly, the lack of curated Class 2 systems available at that time. We discussed the advantage of studying Class I systems (see comment above), and additionally, we also expanded this section of the discussion prompting future studies to do so. See text lines 206–211: “Future studies should investigate whether our findings also apply to Class 2 CRISPR-Cas systems as more data becomes available. This will also afford the opportunity to compare and contrast these two classes. Though Class 2 systems may be currently under-represented, and may actually occur at low frequencies, metagenomics surveys underway, and sampling expeditions aimed at finding new microbial diversity may encompass new Class 2 systems, especially given the desire to find new single effector nucleases.”*

Similarly, are the differences in array lengths and distributions between types and subtypes significant? From the numbers in Table 1, it looks like some could be. If so, it would useful to assess statistically and then, perhaps, consider the biological underpinnings.

Authors’ response: *We thank the reviewer for their suggestion. We thus carried out additional statistical analyses to investigate this and now report that indeed some of these differences are statistically significant. This is discussed in the results section, lines 90–100, where we tested difference between both main types and main subtypes within Type I. We now state: “Our interest also turned to the statistically significant differences that may exist between Type I and Type III systems, as well as between the major four subtypes within Type I systems. We first tested the null hypothesis that there is not a difference between the means for Type I and Type III CRISPR array size. We immediately see from our descriptive statistics that Type I and Type III vary in sample size, so we performed a 2-sample t-test with unequal variances, as not to assume unequal variance. Our result showed a p-value of 0.098, and hence we fail to reject our null hypothesis and conclude that there indeed is no difference when it comes to the mean array sizes of Type I and Type III CRISPRs. Next, we decided to perform the Kruskal-Wallis test, to test our null hypothesis that the subtypes come from identical populations, without making any assumptions about the underlying populations. Our results indicated, with a p-value < 0.001, that we reject our null hypothesis and thus conclude that there are statistically significant differences between the four subtypes, in that they do not come from identical populations.”*

### Reviewer’s report 2: Uri Gophna (Tel Aviv University)

This interesting and timely survey of Class I CRISPR systems, reveals several interesting new properties of these systems including biased genomic locations, and a tendency for systems with multiple arrays to have more spacers at the remote arrays than in the cas-proximal array.

"the geometric distribution was the better fit compared to Poisson. The parameter estimates for geometric and Poisson respectively were 0.02546984 and 38.27681. Both curves were plotted against the histogram and it was immediately apparent that the geometric curve was better suited." - this needs to be re-written providing R or R-squared values for the distributions and minimizing the digits after the decimal point to 3 or 4, throughout the paper.

Authors’ response: *Typically, R-squared values get included when comparing fitted models on training and test datasets. In our case, we are solely assessing one variable (rather than multiple variables) to determine which of two types of distributions best fits to (rather than models) the observed data. Thus, rather than include R-squared values, we performed statistical tests to compare the fit of each distribution, and report parameter estimates accordingly. Specifically, we performed a goodness of fit test to compare and contrast the ability of either distribution type to match the observation. We also wanted to mention that we appreciate input about minimizing digits after a decimal point and thus revised our values throughout the manuscript, with a maximum of 3 digits. See for instance new numbers line 117 and* Table 2.

"Diagnostically, it was necessary to conduct a K-S test as well as plot a cumulative curve for the observed data and candidate distributions. The null hypothesis for the test was that the sample data was drawn from the theoretical geometric distribution since we conducted a one-sample K-S test" - these sentences need to be rewritten.

Authors’ response: *We thank the reviewer for their clarity suggestions and altered the text to: “Diagnostically, we conducted a K-S test and plotted a cumulative curve for the observed data and candidate distributions. We assessed two candidates, the Poisson and the Geometric distributions. The null hypothesis for the one-sample K-S test was that the sample data was drawn from a theoretical geometric distribution. The other null hypothesis was that the sample data was drawn from a theoretical Poisson distribution. The resulting p-value for the former null hypothesis was 0.048 and because the sample is so large with 811 observations, there is a lot of power to detect even some of the smallest differences.” See lines 120–125*.

“Mechanistically, we speculate that this may be correlated to genome-wide processes that could play critical roles in the expansion and maintenance of CRISPR arrays, as well as biochemical processes involved in the new spacer acquisition step during CRISPR immunization, such as chi sites, chromosomal replication and DNA repair processes [20].” Since the authors are speculating about mechanisms, one expects more concrete and detailed hypotheses here - for example selective pressures that minimize delays in replication of the leading strand/collisions when spacers are inserted, or the potential difficulties in replicating the CRISPR locus error-free despite multiple repeats and secondary structure that can have an effect when DNA is in single-stranded form and hence cause CRISPRs to have specific strand biases. Chi sites are important, but many genomes that contain Class I CRISPRs do not have Chi sites or homologs of RecBCD.

Authors’ response: *We thank the reviewer for their insightful suggestion and added a new paragraph to that section accordingly, to speculate on processes involved, lines 157–167. “For instance, it is possible that selective pressure may incentivize the minimization of delays in replication of the leading strand, and select for the absence of collisions and stereo-physical hindrance between the replication machinery and the Cas proteins at the boundaries of CRISPR arrays. This may be most appli-cable to the leader end of CRISPR arrays, where integration occurs and transcription of pre-crRNAs and processing of crRNAs is initiated, and the highest. Furthermore, there are likely two structural challenges inherent to CRISPR loci and CRISPR repeats, since the former contains heavily repeated stretches of DNA (by nature), and the latter is partially palindromic. These repeats and secondary structure therefore pose structural challenges that may be further compounded by multiple interactions with various Cas proteins involved in array maintenance, replication, and expansion. Thus, it is possible that biases in location and orientation of CRISPR loci may reflect selection for the minimization of structural and processing disruptions”*.

#### Minor issues

"for Class I and the two major types for the relationship between the size of the CRISPR closest to the cas genes and the number of CRISPRs in a genome" - this could be more clearly stated.

as "... the number of spacers in the CRISPR array that is closest to the cas genes and the number of arrays in the genome)".

Authors’ response: *This suggestion was accordingly inserted into the manuscript, lines 175–176.*

“and even between enriched subtypes” - should be “well-represented subtypes”.

Authors’ response: *This correction was made in the text line 195*.

## Additional files

Additional file 1: Goodness of fit. Probability plots confirming that overall, class I bacterial chromosome CRISPR arrays tend to follow a geometric distribution. A simulated data set is shown for comparison. (PNG 32 kb)

Additional file 2: CRISPR array occurrence on chromosomes. Polar plots displaying frequency of CRISPRs along a chromosome based on standardization of the location of those arrays differentiated on type and subtype for both positive (blue) and negative orientation (red). (PNG 191 kb)

## Abbreviations

CRISPR: Clustered Regularly Interspaced Short Palindromic Repeats
Cas: CRISPR associated
CRISPR-Cas: Clustered Regularly Interspaced Short Palindromic Repeats - CRISPR associated

## References

[1] Barrangou R, Fremaux C, Deveau H, Richards M, Boyaval P, Moineau S (2007). CRISPR provides acquired resistance against viruses in prokaryotes. Science.

[2] Brouns SJ, Jore MM, Lundgren M, Westra ER, Slijkhuis RJ, Snijders AP (2008). Small CRISPR RNAs guide antiviral defense in prokaryotes. Science.

[3] Marraffini LA, Sontheimer EJ (2008). CRISPR interference limits horizontal gene transfer in staphylococci by targeting DNA. Science.

[4] Garneau JE, Dupuis MW, Villion M, Romero DA, Barrangou R, Boyaval P (2010). The CRISPR/Cas bacterial immune system cleaves bacteriophage and plasmid DNA. Nature.

[5] Barrangou R (2015). Diversity of CRISPR-Cas immune systems and molecular machines. Genome Biol.

[6] Burstein D, Harrington LB, Strutt SC, Probst AJ, Anantharaman K, Thomas BC (2017). New CRISPR-Cas systems from uncultivated microbes. Nature.

[7] Jinek M, Chylinski C, Fonfara I, Hauer M, Doudna JA, Charpentier E (2012). A programmable dual-RNA-guided DNA endonuclease in adaptive bacterial immunity. Science.

[8] Cong L, Ran FA, Cox D, Lin S, Barretto R, Habib N (2013). Multiplex genome engineering using CRISPR/Cas systems. Science.

[9] Barrangou R (2016). Doudna, JA applications of CRISPR technologies in research and beyond. Nat Biotechnol.

[10] Makarova KS, Wolf YI, Alkhnbashi OS, Costa F, Shah SA, Saunders SJ (2015). An updated evolutionary classification of CRISPR-Cas systems. Nat Rev Microbiol.

[11] Makarova KS, Aravind L, Wolf YI, Koonin EV (2011). Unification of Cas protein families and a simple scenario for the origin and evolution of CRISPR-Cas systems. Biol Direct.

[12] Makarova KS, Haft DH, Barrangou R, Brouns SJ, Charpentier E, Horvath P (2011). Evolution and classification of the CRISPR-Cas systems. Nat Rev Microbiol.

[13] Makarova KS, Wolf YI, Koonin EV (2013). The basic building blocks and evolution of CRISPR-CAS systems. Biochem Soc Trans.

[14] Makarova KS, Koonin EV (2015). Annotation and classification of CRISPR-Cas systems. Methods Mol Biol.

[15] Makarova KS, Zhang F, Koonin EV. SnapShot: class 1 CRISPR-Cas systems. Cell 2017;168:946-946 e941. 10.1016/j.cell.2017.02.018 28235204

[16] Makarova KS, Zhang F, Koonin EV. SnapShot: class 2 CRISPR-Cas systems. Cell 2017;168:328-328 e321. 10.1016/j.cell.2016.12.038 28086097

[17] Mohanraju P, Makarova KS, Zetsche B, Zhang F, Koonin EV, van der Oost J (2016). Diverse evolutionary roots and mechanistic variations of the CRISPR-Cas systems. Science.

[18] Koonin EV, Krupovic M (2015). Evolution of adaptive immunity from transposable elements combined with innate immune systems. Nat Rev Genet.

[19] Krupovic M, Makarova KS, Forterre P, Prangishvili E, Koonin EV (2014). Casposons: a new superfamily of self-synthesizing DNA transposons at the origin of prokaryotic CRISPR-Cas immunity. BMC Biol.

[20] Levy A, Goren MG, Yosef I, Auster O, Manor M, Amitai G (2015). CRISPR adaptation biases explain preference for acquisition of foreign DNA. Nature.

[21] R Core Team. R: a language and environment for statistical computing. R Foundation for Statistical Computing, Vienna, Austria. 2016 https://www.R-project.org/. Accessed July 2016.

[22] Wickham H. readxl: Read Excel Files. R package version 0.1.1. 2016 https://CRAN.R-project.org/package=readxl Accessed July 2016.

[23] Wickham H (2011). The split-apply-combine strategy for data analysis. J Stat Softw.

[24] Grissa I, Vergnaud G, Pourcel C (2007). The CRISPRdb database and tools to display CRISPRs and to generate dictionaries of spacers and repeats. BMC Bioinformatics.

[25] Benson DA, Canavanaugh M, Clark K, Karsch-Mizrahi I, Lipman DJ, Ostell J, Sayers EW (2013). GenBank. Nucleic Acids Res.

[26] Delignette-Muller ML, Dutang C (2015). Fitdistrplus: an R package for fitting distributions. J Stat Softw.

[27] Rice J (2006). Mathematical statistics and data analysis.

[28] Scholz FW (1985). Maximum likelihood estimation. Encyclopedia of statistical sciences.

[29] Arnold TB, Emerson JW (2011). Nonparametric goodness-of-fit tests for discrete null distributions. R J.

[30] Wickham H (2007). Reshaping data with the reshape package. J Stat Softw.

[31] Wickham H (2009). ggplot2: elegant graphics for data analysis.

[32] Lemon J (2006). Plotrix: a package in the red light district of R. R-News.

[33] Myers JL (2003). Well AD Research Design and Statistical Analysis.

