# Supplementary figures and images for: On the global CRISPR array behavior in class I systems
Source: Biol Direct. 2017 Aug 29;12:20. doi: 10.1186/s13062-017-0193-2 (PMC5575924; doi:10.1186/s13062-017-0193-2)

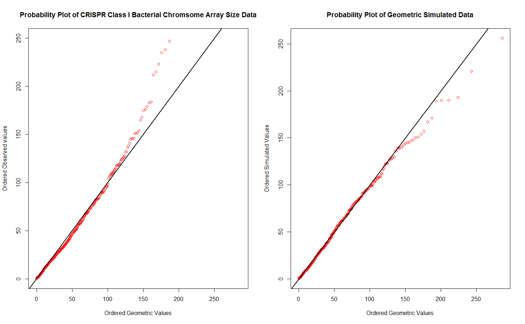

Supplement: Supplementary file 1 — Goodness of fit. Probability plots confirming that overall, class I bacterial chromosome CRISPR arrays tend to follow a geometric distribution. A simulated data set is shown for comparison. (PNG 32 kb) [file 13062_2017_193_MOESM1_ESM.png]

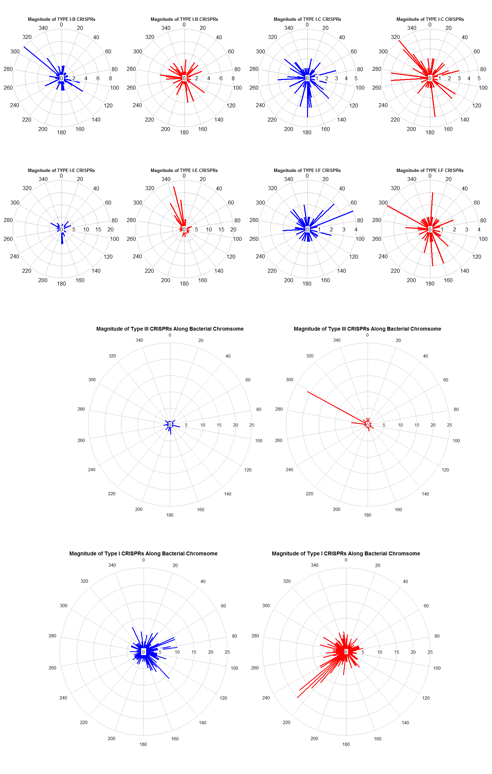

Supplement: Supplementary file 2 — CRISPR array occurrence on chromosomes. Polar plots displaying frequency of CRISPRs along a chromosome based on standardization of the location of those arrays differentiated on type and subtype for both positive (blue) and negative orientation (red). (PNG 191 kb) [file 13062_2017_193_MOESM2_ESM.png]
